# Ionic Liquid Assisted Imprint for Efficient and Stable Quasi-2D Perovskite Solar Cells with Controlled Phase Distribution

**DOI:** 10.1007/s40820-023-01076-8

**Published:** 2023-04-07

**Authors:** Haibin Peng, Dengxue Li, Zongcai Li, Zhi Xing, Xiaotian Hu, Ting Hu, Yiwang Chen

**Affiliations:** 1 Department of Polymer Materials and Engineering, School of Physics and Materials Science, Nanchang University, 999 Xuefu Avenue, Nanchang, 330031 People’s Republic of China; 2 College of Chemistry and Chemical Engineering , Institute of Polymers and Energy Chemistry (IPEC), Nanchang University, 999 Xuefu Avenue, Nanchang, 330031 People’s Republic of China; 3 National Engineering Research Center for Carbohydrate Synthesis/Key, Lab of Fluorine and Silicon for Energy Materials and Chemistry of Ministry of Education, Jiangxi Normal University, 99 Ziyang Avenue, Nanchang, 330022 People’s Republic of China; 4 Peking University Yangtze Delta Institute of Optoelectronics, Nantong, 226010 People’s Republic of China

**Keywords:** Imprint, Two-dimensional perovskite, Phase distribution, Charge transfer, Stability

## Abstract

Although two-dimensional perovskite devices are highly stable, they also lead to a number of challenges. For instance, the introduction of large organic amines makes crystallization process complicated, causing problems such as generally small grain size and blocked charge transfer. In this work, imprint assisted with methylamine acetate were used to improve the morphology of the film, optimize the internal phase distribution, and enhance the charge transfer of the perovskite film. Specifically, imprint promoted the dispersion of spacer cations in the recrystallization process with the assistance of methylamine acetate, thus inhibited the formation of low-n phase induced by the aggregation of spacer cations and facilitated the formation of 3D-like phase. In this case, the corresponding quasi-2D perovskite solar cells delivered improved efficiency and exhibited superior stability. Our work provides an effective strategy to obtain uniform phase distribution for quasi-2D perovskite.

**Supplementary Information:**

The online version contains supplementary material available at 10.1007/s40820-023-01076-8.

## Highlights

- The strategy of imprint assisted with methylamine acetate effectively ameliorates the morphology of the film, optimize the internal phase distribution, and enhance the charge transfer of the perovskite film.
- The combination of imprinting with additive engineering improves the phase distribution within quasi-2D perovskite and promote the carrier transport, resulting in a power conversion efficiency of 18.96% with specific good reproducibility.

**Supplementary Information:**

The online version contains supplementary material available at 10.1007/s40820-023-01076-8.

## Graphical abstract

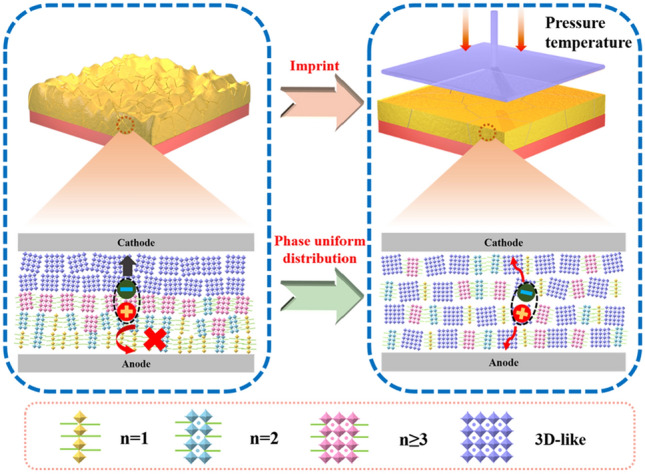

## Introduction

Lead halide perovskites have drawn tremendous attention in the past decade because of their unique properties, such as long charge carrier diffusion length, high light absorption coefficient, and excellent defect tolerance [1]. Although the power conversion efficiency (PCE) of perovskite solar cells (PSCs) has been dramatically improved to around 25.7% recently [2], the instability of PSCs is still an urgent challenge to be solved. Because the light-absorbing material, perovskite, has poor chemical stability and is prone to degrading under UV light, oxygen, humidity, high temperature and other environmental conditions [3, 4]. Until now, various methods have been proposed to improve the long-term stability of PSCs, such as interface engineering, encapsulation, and material designing [5–9] Recently, the two dimensional (2D) perovskites with excellent performance in stability has become one of the most promising candidate for stable PSCs [10–15].

2D perovskites are expected to be a promising alternative to three dimensional (3D) perovskite due to their enhanced environmental stability and greater structural freedom and adjustable band gap [13]. Ruddlesden-Popper (RP) perovskite is one type of 2D perovskites with the general formula (R-NH_3_)_2_(A)_*n*−1_M_*n*_X_3*n*+1_, where R is an alkyl or aromatic moiety, A is a small cation (e.g., methylammonium, MA^+^), M is a divalent metallic cation (such as Pb^2+^) and X is a halide (such as Cl^−^, Br^−^, or I^−^) [9–11]. Their structure consists of inorganic layers of corner-sharing MX_6_^4−^ octahedra sandwiched between long-chain organic cations and the halide anions [17]. The variable “*n*” indicates the number of MX_6_^4−^ layers stacked between two R-NH_3_^+^. Compared with 3D perovskite, 2D-RP perovskite has significant suppression of internal body defects in perovskite due to the internal doping of hydrophobic and bulky long-chain ligands, while the humidity stability is significantly improved [18, 19].

Due to the self-assembly effect formed by hydrogen bonding interactions between insulated organic ammonium cations and inorganic perovskite sheets, the inorganic perovskite sheets are sequentially separated by the organic ammonium cation layers, resulting in an organic–inorganic alternating quantum well structure in 2D-RP perovskite, where the organic ammonium ions act as a quantum barrier and the inorganic perovskite sheets act as a quantum well. As a result of the quantum confinement, 2D-RP perovskite exhibits better stability than 3D perovskite because the ion migration can be significantly suppressed by organic cation layer. In addition, the alternating organic–inorganic layer structure of 2D-RP perovskites provides better energy band tunability for material optimization. Meanwhile, introduction of hydrophobic organic spacer cations in 2D-RP perovskites can passivate the defects caused by the absence of methylamine cations. The special structure and excellent physicochemical properties of 2D-RP perovskite enhance its stability in atmospheric environments [11, 20–22]. Although 2D-RP perovskites possess many advantages, some problems need to be solved urgently if 2D-RP perovskite is employed as light-absorbing material for efficient PSCs. For example, the quantum well structure will also hinder charge transport and increase charge recombination because of the insulation characteristics of organic cations. Moreover, due to the large spatial site resistance of organic spacer cations, the introduction of organic cations complicates the crystallization process of perovskite and makes the morphology of the film difficult to control, which aggravates the non-radiative recombination of 2D-RP perovskite and further affects the photoelectric performance of PSCs [23, 24].

Therefore, various methods, including hot-cast deposition, solvent engineering, and additive engineering have been developed to modulate the crystal growth orientation to facilitate charge transport in 2D-RP perovskite [25–29]. However, the charge transport of 2D-RP perovskites is still unsatisfactory. Therefore, regulating the phase distribution is proposed as a promising approach to improve the charge transport of quasi-2D perovskites [30–32]. For instance, Shao and co-workers employed formamidinum (FA) cations to replace MA cations to substantially increase the portion of 3D-like phase to facilitate charge transport for efficient and stable 2D PSC [33]. Kong et al. employed an additive strategy to reconstruct quasi-2D perovskite structures by the interaction between the strong hydrogen bonding of methane sulfonates and spacer BA cations, leading to improved energy acceptor–donor ratio of 2D perovskite films and enhanced energy transfer [34]. Huang's team formed an intermediate phase of perovskite to make the phase distribution more uniform by introducing molten salt spacer n-butylamine acetate into the precursor solution, resulting in improved photoelectric performance of PSC with PCE of 16.25% [35]. According to the literatures, additive strategies were employed to optimize the phase distribution of 2D perovskites to promote charge transport, but these methods still have some limitations for improving film crystallization and controlling the film morphology. It was reported that the directional compressive stress by imprinting can make perovskite confined crystallization and effectively improve the perovskite crystal quality. For example, simply pressing a hexagonal nanodot array of polyurethane stamps onto a partially dried 3D perovskite interlayer can improve the crystal orientation and the film quality of 3D perovskite [36]. The combination of pressure-induced crystallization and phase distribution modulation strategies may effectively improve the charge transport caused by crystallographic variation, thus increasing the PCE of quasi-2D perovskite devices.

In this study, a combination of imprint and additive strategies was used to prepare quasi-2D perovskite with uniform phase distribution. The imprint can facilitate confinement crystallization and phase rearrangement in quasi-2D perovskite, which can promote the dispersion of spacer cations in the recrystallization process, and thus inhibit the formation of low-n phase induced by the aggregation of spacer cations and impel the formation of 3D-like phase. The ionic liquid methylamine acetate (MAAc) can effectively inhibit the aggregation of colloids in the precursor solution, and thus further promote the recrystallization in the process of imprint. Therefore, the combination of these two strategies is expected to improve the phase distribution within quasi-2D perovskite and promote the carrier transport, which will be verified later.

## Experimental Section

### Materials

N, N-dimethylformamide (DMF, 99.8% purity), dimethyl sulfoxide (DMSO, 99.9% purity), acetonitrile (99.8% purity), chlorobenzene (CB, 99.8% purity), 4-tert-butyl pyridine (TBP) and CH3NH3I (MAI, >98% purity) were purchased from Sigma-Aldrich and used as received without further purification. Lead iodide (PbI2, 99.9985% purity), tin(IV) oxide (15% in H_2_O colloidal dispersion liquid) and lithium bis(trifluoromethylsulfonyl) imide (Li-TFSI, >98% purity) were purchased from Alfa Asear. 2,2',7,7'-Tetrakis [N, N -di(4-methoxyphenyl) amino]-9,9'-spirobifluorene (spiro-MeOTAD, 99% purity) was purchased from Luminescence Technology Crop. Methylammonium Acetate (MAAc, 99.5% purity) was purchased from Xi’an p-OLED Corp. Trimethoxy (1H,1H,2H,2H-heptadecafluorodecyl) silane (FAS, 98% purity) was purchased from Innochem.

### Preparation of Anti-Sticking Layer

First, clean silicon wafers are placed in an evaporation dish and 200 uL FAS is dropped in the dish. finally, FAS was deposited on top of Si stamp by evaporation in high vacuum for 3 h.

### Solar Cell Fabrication

ITO-coated glass substrates with a sheet resistance of 15 Ω sq^–1^ was consecutively cleaned in an ultrasonic bath containing detergent, acetone, deionized (DI) water, and ethanol for 10 min in each step and then dried by nitrogen. Prior to film deposition, the substrate was treated by UV light for 10 min. SnO_2_ precursor was prepared by mixing the SnO_2_ colloidal solution with DI water by a ratio of 1:3. SnO_2_ was spin coated on the ITO glass at 3,000 rpm for 30 s, annealing at 150 °C for 30 min. The precursor solution was prepared by adding PEAI, PbI_2_, MAI (2:50:49) and 5 uL mL^−1^ MAAc into the mixture of DMF and DMSO (9:1). Then the perovskite solution was deposited on the SnO_2_ surface at 4,000 rpm for 30 s in N_2_ atmosphere and dried 100 °C. Simultaneously, 150 μL CB solution was fastly drop-casted in nine seconds. At a pressure of 3 MPa and a preset temperature (150 °C), the perovskite film is placed on the silicon wafer for imprint for 10 min in air. After cooling down, 40 uL Spiro-OMeTAD solution was spun with a ratio of 4,000 rpm for 30 s. The Spiro-OMeTAD solution was prepared by adding 72.3 mg spiro-OMeTAD in the solvent (CB 1 mL, 4-tertbutylpyridine 28.8 uL, Li-TFSI acetonitrile solution 17.5 uL, 520 mg mL^–1^). Finally, ≈100 nm Ag was deposited through a shadow mask at a pressure of 7 × 10^−4^ Pa in a vacuum.

### Characterization

Keithley 2400 was used to characterize the current density–voltage (*J*-*V*) curves. The currents were measured under a solar simulator (Enli Tech, 100 mW cm^−2^, AM 1.5 G irradiation). The active area of the device and the area of the shadow mask are 0.04 cm^2^. The light intensity was calibrated by means of a KG-5 Si diode with a solar simulator (Enli Tech, Taiwan). Devices were stored and tested in the nitrogen-filled glovebox. Scanning electron microscopy (SEM) images were conducted on SU8020 scanning electron microscope operated at an acceleration voltage of 5 kV. X-ray diffraction (XRD) spectra were carried out by using X-ray diffractometer (Rigaku D/Max-B). The ultraviolet–visible (UV–Vis) spectra were characterized on UV-2600 spectrophotometer (SHIMADZU). The steady-state photoluminescence (PL) and time-resolved photoluminescence (TRPL) spectra were recorded by an Edinburgh instruments FLS920 spectrometer (Edinburgh Instruments Ltd.). X-ray photoelectron spectroscopy (XPS, Thermo Scientific ESCALAB 250Xi) was used for binding energy and element distribution analysis. Electrical impedance spectroscopy (EIS) of the devices was performed in a frequency range from 1 MHz to 10 mHz using Zahner electrochemical workstation at an applied bias equivalent to the open-circuit voltage of the cell under 1 sun illumination. The trap density of states was deduced from the angular frequency dependent capacitance. The currents were measured under 100 mW cm^−2^ simulated AM 1.5 G irradiation (Abet5 Solar Simulator Sun2000). The water contact angle was measured at a Krüss DSA100s drop shape analyser.

## Results and Discussion

### Fabrication and Characterization of the Quasi-2D Perovskite Films

**Fig. 1:**
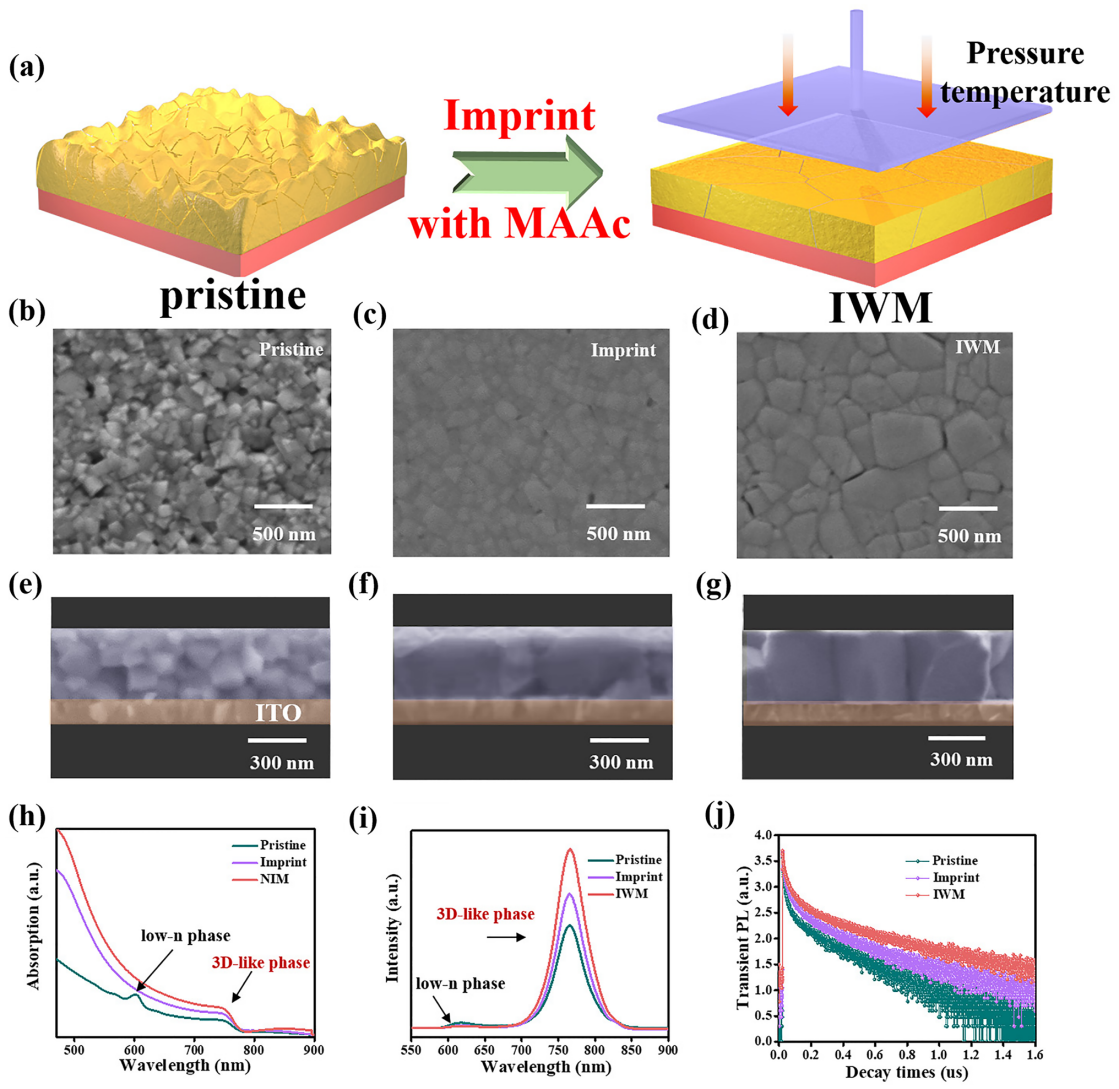
**a** Schematic diagram of morphology changes after IWM treatment. **b-g** The top-view and cross-sectional SEM images of: **b**, **e** Pristine; **c**, **f** Imprint and **d**, **g** IWM. **h** UV–vis absorption spectra. **i** Steady-state PL spectra. **j** TRPL spectroscopies

The quasi-2D perovskite (PEA_2_MA_49_Pb_50_I_151_) precursor solution was prepared by dissolving methylammonium iodide (MAI), phenylethylammonium iodide (PEAI), and lead iodide (PbI_2_) in N, N-dimethylformamide (DMF)/dimethyl sulfoxide (DMSO) binary solvent. In the preparation process, the precursor solution with the addition of MAAc was spin-coated to prepare perovskite films. And then quasi-2D perovskite films with MAAc were imprinted by using a planar silicon template with cross-linked silanes anti-adhesive layer at a given pressure and temperature (Fig. 1a). During spin-coating process and high temperature embossing, MAAc evaporates as a by-product due to its low boiling point [35]. For convenient, the sample “imprint perovskite film with MAAc” is abbreviated to “IWM”. As proved in our previous work, recrystallization happens in the imprinting process, which results in larger grains and compact film during the imprint process [37].

As depicted in Fig. 1b-g, the pristine film showed some cracks and pinholes, and the grain size of the pristine film was mostly around 100 nm (Fig. 1b). While the imprint perovskite films appear more compact and uniform, and the grains of quasi-2D perovskite have significantly enlarged to 150–200 nm (Fig. 1c). However, there were still some small grains in imprint film. So, ionic liquid MAAc was employed to address this issue. MAAc can inhibit colloid aggregation [35], The addition of MAAc effectively inhibits the aggregation of colloids, which can significantly improve the crystallization process of quasi-2D perovskite and make the grains larger. After introducing MAAc, IWM film displayed a dense and smooth surface morphology and the grain size of IWM film was further increased to around 400 nm. And the grain size of IWM films remained around 400 nm after our repeated experiments, showing excellent reproducibility (Fig. 1d). The above crystal morphology can be further confirmed by the cross-sectional SEM in Fig. 1e-g. Compared to the pristine perovskite film with disorderly small crystals (Fig. 1e), the internal crystal quality of perovskite films was significantly improved after imprint treatment (Fig. 1f). Moreover, IWM perovskite is vertical growth to the substrate (Fig. 1g), which is beneficial to the charge transfer and thus improve the short-circuit current density (*J*_SC_).

The UV–vis absorption spectra of pristine, imprint and IWM quasi-2D perovskite films (*n* = 50) are showed in Fig. 1h. It can be found that there were two obvious excitonic peaks corresponding to a distribution of perovskite phases with low-n phases and 3D-like phase for pristine quasi-2D perovskite films, indicating the existence of multiple perovskite phases in the pristine films. However, no obvious excitonic absorption peaks were observed for the imprint film, suggesting that the formation of low-n phase was suppressed by imprint treatment. And the absorption intensity of imprint film is enhanced after imprint, which may be attributed to the transformation from low-n phase to 3D-like phase. Similarly, for IWM film, there was no obvious absorption peak in the short-wave range and the absorption intensity of IWM film was further improved. This phenomenon can be ascribed to the increased 3D-like phases induced by IWM in quasi-2D perovskite film, which means that the MAAc is conducive to the formation of 3D-like phases, resulting in stronger absorption of quasi-2D perovskite film.

To further understand the phase distribution and charge dynamics of pristine, imprinted and IWM quasi-2D perovskite films, time-resolved PL (TRPL) spectroscopies and steady-state PL were tested. The steady state PL on the front side of the film was tested with an excitation light wavelength of 500 nm. As shown in Fig. 1i, the major PL peak of the IWM and imprint film are located at 765 nm, which is related to the 3D-like phases. In comparison, besides the major peak, another obvious shoulder peak at ~ 620 nm was observed in the pristine film, suggesting the formation of low-n phases in pristine quasi-2D perovskite film, which is consistent with the UV–vis absorption data discussed above. Besides, the IWM and imprint film exhibited stronger PL intensity than that of the pristine film, indicating that the perovskite film has fewer defects with higher crystal quality, leading to reduced non-radiative recombination. This was further demonstrated by the TRPL measurements (Fig. 1j). To obtain a detailed fitted carrier lifetime, a single exponential decay function (Y = A_1_exp(− t/*τ*_1_) + A_2_exp(− t/*τ*_2_)) was fitted. TRPL uses an excitation wavelength of 490 nm to excite the front side of the film and obtain the carrier lifetime of different films. The pristine film showed carrier lifetimes of 98.4 ns for *τ*_ave_. The carrier lifetimes of the IWM and imprint film were calculated to be 182.3 and 131.6 ns for *τ*_ave_, respectively, indicating the reduction of defects within the quasi-2D perovskite, leading to a longer diffusion length of the excitons in 2D perovskite and efficient exciton extraction between 2D perovskite and the charge transport layer. The significantly increased lifetime for the imprint film could be attributed to its improved crystallinity quality with less defects, leading to suppressed nonradiative recombination, which was further confirmed by the calculated defect density in perovskite films as discussed below. Furthermore, for IWM film, the carbonyl group in the ionic liquid MAAc can interact with the Pb^2+^ in the precursor solution, thus enabling a homogeneous distribution of colloids in the precursor. Thus, uniformly distributed colloids can suppress the gradient crystallization of quasi-2D perovskite, which brings about improved crystal quality. Pb 4*f* chemical state of XPS was tested to further confirm the interaction between carbonyl group of MAAc and Pb^2+^ in IWM quasi-2D perovskite (Fig. S1). The Pb 4*f*_5/2_ and Pb 4*f*_7/2_ peaks of the pristine films were located at 142.2 and 138.2 eV, respectively. After treatment with MAAc, the Pb 4*f*_5/2_ (142.4 eV) Pb 4*f*_7/2_ (138.4 eV) signal shifts to a lower binding energy by about 0.3 eV. The negative shift in the Pb 4*f* binding energy indicates a reduction in the cationic charge of the Pb^2+^, as the carbonyl group gifts the lone electron pair of oxygen atom to the empty 6*p* orbital of the Pb^2+^ cation. Generally, quasi-2D perovskite precursor solutions usually contain colloidal particles with random-size distribution, which can act as nucleation sites to bring about poor crystal morphology of quasi-2D perovskite [38, 39]. It is demonstrated that MAAc interacts with Pb^2+^ in quasi-2D perovskite and this interaction Pb^2+^ to a more homogeneous colloidal distribution in the precursor solution by XPS test, which contributes to the formation of a more homogeneous composition during the crystallization process of quasi-2D perovskites.

Meanwhile, as shown in Fig. S2, the dynamic light scattering of quasi-2D perovskite precursor solutions with and without MAAc were tested, respectively. It shows that the colloidal size is significantly suppressed after the addition of MAAc, which will facilitate the crystallization of quasi-2D perovskite. This narrowed size distribution may be due to the strong interaction between Ac^−^ and Pb^2+^, which prevents the aggregation of colloids. To further demonstrate the strong interaction between the carbonyl group in MAAc and the Pb^2+^ in precursor solutions, total reflection fourier-transform infrared spectra (FTIR) of PbI_2_ with and without MAAc were also tested, as shown in Fig. S3. The asymmetric and symmetric stretching vibration peaks of Ac^−^ are located at 1557 and 1402 cm^−1^, respectively. The shifts of corresponding stretching vibration peaks proved the coordination of the carbonyl group in MAAc with the Pb^2+^ in quasi-2D perovskite [35]. Consequently, MAAc can inhibit the aggregation of colloids and improve the crystal quality of quasi-2D perovskite.

To further investigate the synergistic effect of imprint and MAAC on the crystallinity and orientation of quasi-2D perovskite, X-ray diffraction (XRD) of pristine, imprint and IWM perovskite films were tested (Fig. S4). It can be observed that the pristine films showed a low crystallinity, and the intensity of the diffraction peaks of the films was enhanced after imprint, and the intensity of the diffraction peaks was further enhanced after the addition of MAAc. Meanwhile, the peak intensity of 3D perovskite (110) was significantly enhanced, which represented the increase of 3D-like phases to some extent. The above results show that imprint can improve the crystallization quality of quasi-2D perovskite film, where IWM film has the largest and most regular grains, and the cross-sectional view also shows that IWM film has more complete grains, and these characteristics enable enhanced charge transport and surface hydrophobicity of the film. And the ratio of (202) and (111) crystalline planes was calculated to manifest the vertical orientation of quasi-2D perovskite. It can be seen that the XRD intensity ratio of (202)/I(111) of the IWM perovskite film is 0.59 and imprinted films is 0.57, which are larger than that of the pristine films, indicating that the internal layered phase of the imprinted perovskite film tends to grow perpendicular to the substrate, which facilitates effective charge transport in the device [40]. To further investigate the effect of imprinting temperature on the crystallinity of perovskite films, XRD was tested at different imprint temperatures for the same imprint time. As shown in Fig. S5, the crystallinity of perovskite films improved with the increase of imprinting temperature.

**Fig. 2:**
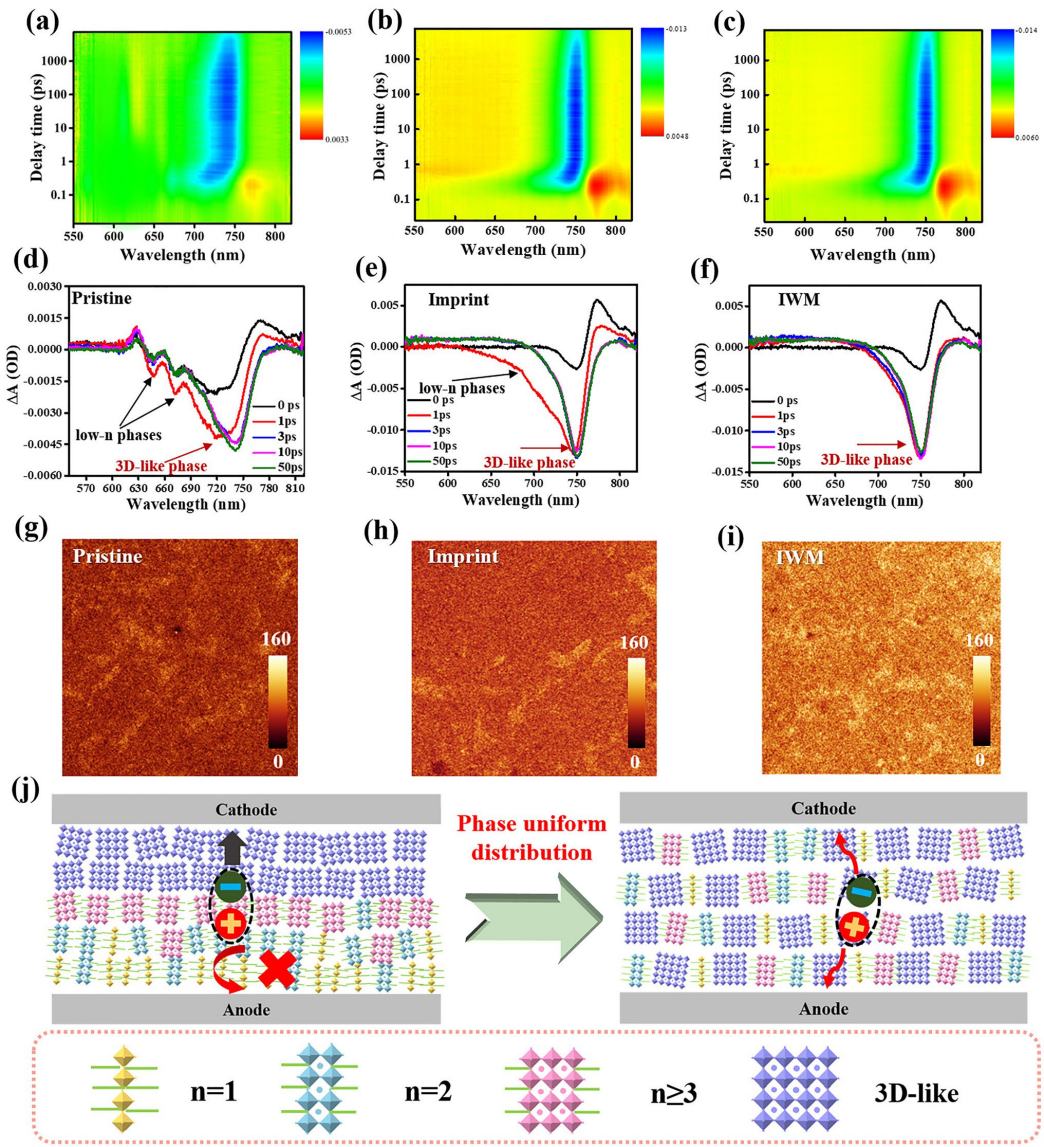
**a−c** Transient absorption (TA) color maps of **a** quasi-2D perovskite films pristine, **b** imprint, and **c** IWM. **d−f** TA spectra at various delay times of: **d** pristine; **e** imprint and **f** IWM. **g−i** PL confocal microscope spectra of: **g** pristine; **h** imprint and **i** IWM. (region size: 20 × 20 mm). **j** Schematic diagrams of the uniform phase distribution as conductive channel

To study the phase distribution and energy transfer process of the perovskite films, femtosecond transient absorption (TA) was tested [33, 41] (Fig. 2a–f). The pristine quasi-2D perovskite film featured ground-state bleaching (GSB) peaks around ≈645, 672 and 750 nm, assigning to the weak low-n phases and 3D-like phase, respectively (Fig. 2a, d), and indicating the existence of low-n phases in quasi-2D perovskites even with high n value (n = 50). Usually, quasi-2D perovskite films deposited by solution method consist of multiple 2D perovskite phases with different n-values, and these 2D perovskites with different n-values are randomly arranged to form quantum wells with different widths, which leads to severe energy disorder. Generally, quasi-2D perovskite films deposited by solution methods usually contain multiple 2D perovskite phases with different n-values, leading to severe energy disorder [42–44]. Meanwhile, the charge transport in the quasi-2D perovskite was hindered due to the dielectric mismatch induced by the large organic cations of low-n phases, which will seriously affect the photoelectric performance of PSCs. Conversely, as shown in Fig. 2b, e, the imprint film only exhibited strong GSB signals at around 750 nm, accompanied with the fading of the GSB peaks around ≈645, 672 nm, implying the dominated 3D-like phases and the suppressed low-n phase in the quasi-2D perovskite film. The reason is that the slow recrystallization driven by imprint can induce the rearrangement of organic spacer cations in quasi-2D perovskite [45], and thus inhibit the formation of low-n phase induced by the aggregation of spacer cations. Due to the rearrangement of spacer cations, the low-n phases actually tend to convert to the 3D-like phase. Furthermore, as shown in Fig. 2c, f, the IWM film only exhibited stronger GSB signals at around 750 nm with no obvious GSB signals of low-n phase, which indicated that most of low-n phase was converted to the 3D-like phase. It is because that the ionic liquid MAAc can effectively inhibit the aggregation of colloids in the precursor solution, and thus further promote the recrystallization in the process of imprint. Therefore, imprinting assisted by MAAc can make the more dispersed distribution of organic cations, and thus inhibiting the formation of low-n phase induced by the aggregation of spacer cations and impelling the formation of 3D-like phase. The aggregated 2D phase disappears and is uniformly distributed throughout the perovskite layer, so the disappearance of the 2D phase and the simultaneous increase of the 3d-like phase were observed in the characterization. The abundant 3D-like phases in IWM quasi-2D perovskite films provide efficient channels for carrier extraction, which will lead to higher *J*_SC_ in quasi-2D PSCs. Besides, the decay of GSB peaks assigning to low-n phases is accompanying by the rise of GSB peak at 3D-like phases, indicating a cascade energy transfer from the low-n to 3D-like phases [34].

Then the mapping of PL peak position was used to further study the phase transition of quasi-2D perovskite film (Fig. 2g–i). The brightness intensity of the image in fluorescence confocal is related to the light intensity of the light at 760 nm after excitation, and the light at 760 nm represents the excitation wavelength of the 3D-like phase, therefore the intensity and distribution of fluorescence confocal represent the content and distribution of the 3D-like phase. The brightness of the pristine films was the lowest, and the distribution of brightness intensity varies significantly, indicating heterogeneous phase distribution. By comparison, the imprint film was brighter with improved brightness difference, indicating that 3D-like phase exists more widely. The IWM films revealed brightest fluorescence, which is uniformly distributed on the surface of the film, suggesting that the IWM films possess uniform phase distribution and maximum content of 3D-like phase [46–48]. Combined with the previous TA test, it can be inferred that the imprint treatment can exactly boost the conversion from low-n phase to 3D-like phase. By adding MAAc, the low-n phase almost disappeared and turned into 3D-like phase, suggesting that IWM efficiently realized the change from low-n phase to 3D-like phase. In consequence, the widespread 3D-like phase could provide efficient channels for carrier extraction, resulting in enhanced charge transfer in the quasi-2D perovskite film (Fig. 2j).

### Photovoltaic Performance of Quasi-2D PSCs

**Fig. 3:**
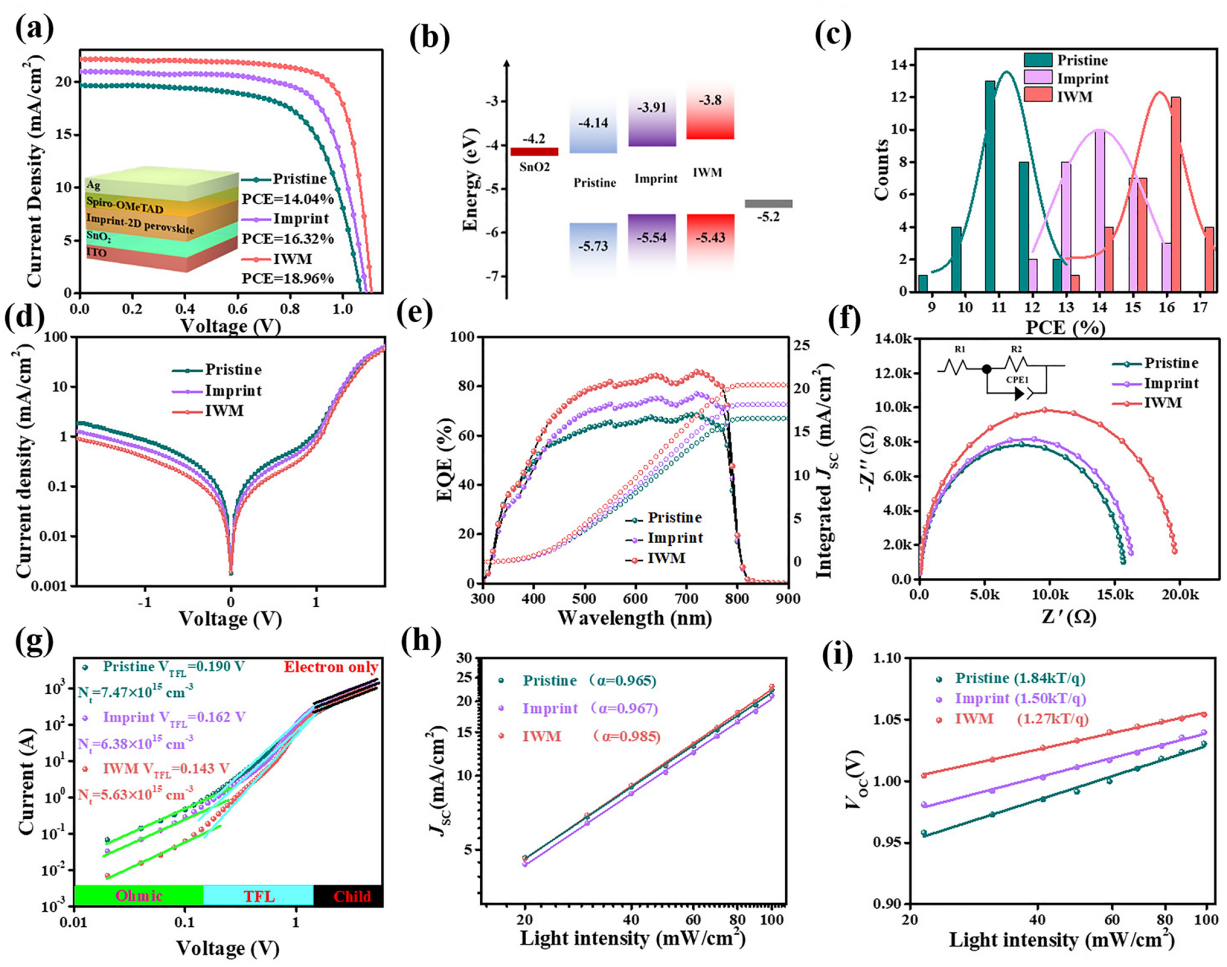
**a** *J*-*V* curve and efficiency data of the pristine, imprint and IWM PSCs. The insert picture is the device architecture. **b** Schematic energy levels of pristine, imprint, and IWM perovskites. **c** Reproducibility of PCE. **d** Dark *J-V* curves of the pristine, imprint, and IWM PSCs (n = 50). **e** External quantum efficiency of pristine, Imprint, and IWM PSCs. **f** EIS of the pristine, imprint, and IWM PSCs (n = 50). **g** Dark *J*-*V* curves for pristine, imprint and IWM based electron-only devices. **h−i** Light intensity dependence of *J*_SC_, *V*_OC_

Next, the n–i–p planar heterojunction PSCs based on pristine, imprint and IWM perovskite film with an architecture of glass/ITO/SnO_2_/perovskite/spiro-OMeTAD/Ag were fabricated. The current density voltage (*J*-*V*) curves for the standard AM 1.5G illumination were shown in Fig. 3a. The pristine (n = 50) based device has a PCE of 14.04%, a *J*_SC_ of 19.70 mA cm^2^, an open circuit voltage (*V*_OC_) of 1.06 V, and a fill factor (FF) of 66.69%. In contrast, the PCE of the imprinted (n = 50) device increased significantly to 16.32% with a *V*_OC_ of 1.08 V, *J*_SC_ of 20.96 mA cm^−2^, and FF of 71.47%. Encouragingly, the IWM PSC delivered a champion PCE of 18.96% with a *V*_OC_ of 1.10 V, *J*_SC_ of 22.15 mA cm^−2^, and FF of 77.20%. At present, the PCE of this work is close to the highest PCE (20%) of phenylethylammonium-based 2D perovskite devices, showing competitiveness (Table S1). Devices based on imprint and IWM treatment exhibit higher *V*_OC_ and *J*_SC_ compared to pristine devices, which may be due to increased carrier lifetime, lower trap density, and reduced carrier non-radiative recombination losses. The IWM treatment can effectively improve the crystal quality and promote the transition of perovskite from low-n phase to 3D-like phase, so the widespread 3D-like phase could provide efficient channels for charge transfer, resulting in remarkably improved *J*_SC_ in the IWM PSC. The ultraviolet photoelectron spectra (UPS) were carried out to understand the charge transport properties as shown in Fig. S6, the valence band maxima (VBM) and the energy-level alignment of pristine, imprint and IWM perovskite films were shown in Fig. 3b. The maximum value of the imprint and IWM perovskite film valence band is shifted upward compared with that of pristine perovskite film, which could optimize energy level matching, thus suppressing the charge recombination [3, 49–51]. To further demonstrate the photoelectric performance of pristine, imprint and IWM PSC, the steady-state photocurrent and stabilized PCE output under standard AM 1.5G illumination for 400 s by maintaining the voltage at the maximum power points was characterized (Fig. S7). The pristine and IWM devices displayed the stabilized PCE of 13.58% and 18.15%. The stabilized IWM PSCs is closer to the PCE in the *J-V* curve of Fig. 3a, implying that the IWM device has an excellent operational stability in the illuminated environment. Since the IWM can make the quasi-2D perovskite crystallize uniformly, the 3D-like phase inside can be distributed uniformly. Uniform 3D-like phase distribution can act as a solid channel for charge transport, resulting in stable *J*_SC_.

In order to compare the hysteresis phenomenon in the pristine, imprint and IWM PSCs, the forward and reverse scan of devices were tested, as shown in Fig. S8. The imprinted and IWM devices showed negligible hysteresis by comparing to the pristine devices. Besides, the Gaussian distribution of PCE of pristine imprint and IWM devices was statistically characterized (Fig. 3c), showing a higher average PCE and better reproducibility. Figure S9 show the distribution of *J*_SC_, *V*_OC_ and FF for pristine, imprint and IWM devices. It is worth noting that the IWM devices have a narrower distribution in *V*_OC_ and FF, which represents a higher reproducibility of the IWM and imprint devices. In order to further study the charge complexation of different devices, dark J-V curves tests were conducted as shown in Fig. 3d. It is observed that the dark current density of IWM device is reduced compared to pristine and imprint, which indicated that the lower bulk defects in the quasi-2D PSC. Figure 3e shows the external quantum efficiency (EQE) as well as the integrated *J*_SC_ of the pristine, imprint and IWM quasi-2D PSCs. Especially, the integrated *J*_SC_ of 20.84 mA cm^−2^ for the IWM device is in better agreement with the measured value of the J-V curve, while the integrated *J*_SC_ of 17.21 mA cm^−2^ for the pristine devices. Electrochemical impedance (EIS) is an effective test to analyze the composite behavior and interfacial charge transport of photovoltaic devices. The EIS of pristine, imprint and IWM devices were tested as shown in Fig. 3f. The EIS test was performed in a dark environment, keeping the bias voltage at 0.8 V. IWM devices have lower Rtr and higher Rrec compared to the pristine, which indicates that the devices have better charge transfer and less carrier recombination, verifying the synergistic effect of IWM on promoting the growth of 3D-like phases [52].

To test the trap state density (*N*_t_) of the three devices, pristine, imprint and IWM PSCs of ITO/SnO_2_/PVK/PCBM/Ag configurations were prepared for purely electronic devices (Fig. 3g). The trap density of states of pristine, imprint and IWM electron-only devices was calculated as follows equation *N*_t_ = (2*ε*_0_ *ε*_r_ *V*_TFL_)/(qd^2^). As a result, the *N*_t_ is calculated to be 7.47 × 10^15^ cm^−3^ for the pristine and 6.38 × 10^15^ and 5.63 × 10^15^ cm^−3^ for the imprint and IWM, respectively. The electron trap densities of imprint and IWM devices are greatly reduced compared to the pristine sample, which is consistent with the longer carrier lifetime and diffusion length. Thus, IWM and imprint treatment can effectively reduce the trap state and restrain the nonradiative recombination in the device, so that the reduction of the trap density in the film. In addition, the light intensity (*P*_light_) related* J*_SC_ and can help to understand the charge recombination in the device. Figure 3h plots the *J*_SC_ as a function of incident light intensity *P* according to the equation *J*_SC_ ∝ *P*^α^. The deviation from the ideal factor α = 1 codifies the bimolecular complexation of the device, so that higher values of α indicate having lower molecular complexation due to defects [53, 54]. It can be found that the power factor α of IWM device is closer to 1 compared to imprint and pristine devices, indicating that the recombination reduced caused by defects after the IWM treatment. Based on the relationship between* V*_OC_ and the logarithm of the incident light intensity* P*, the ideal factor n can be determined by the slope nk_B_T/q. The slope of the IWM device is the smallest (Fig. 3i), confirming the reduction of trap-assisted recombination. This is because MAAc can adjust the distribution of solution colloids and reduce the internal defects caused by perovskite in the crystallization process, while the imprinting further reduces the internal bulk defects, resulting in lower trap-assisted recombination.

### Stability of Quasi-2D Perovskite Films and Devices

**Fig. 4:**
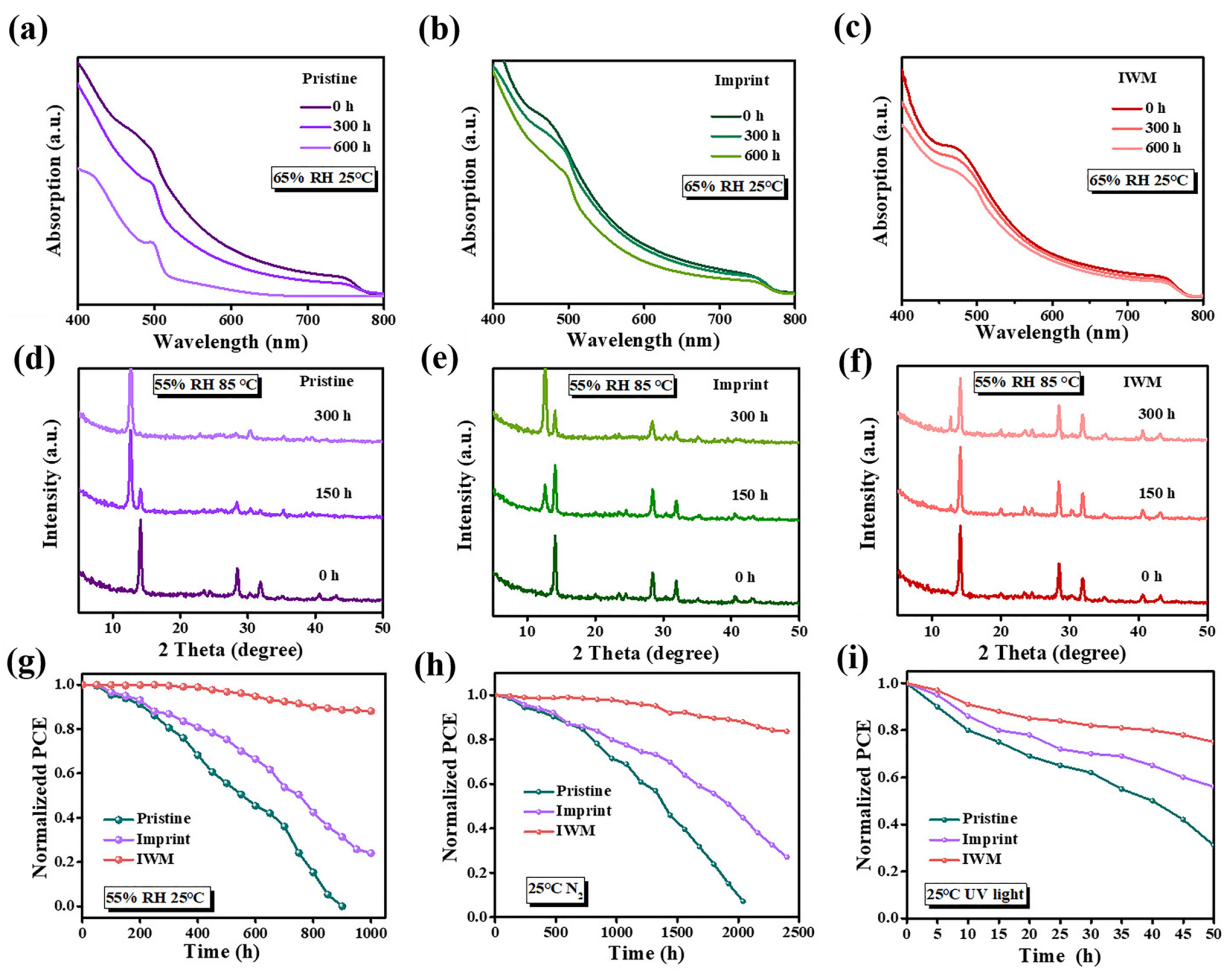
**a–c** UV–vis absorption spectra of pristine, imprint, and IWM perovskite films before and after aging under about 65% RH 25 °C. **d–f** XRD patterns of pristine, imprint, and IWM perovskite films before and after aging at 85 °C in air with 55% RH. **g** Normalized PCE variation curves of unsealed pristine, imprint, and IWM devices at 25 °C in air with 55% RH. **h** Standardized PCE of unsealed pristine, imprint, and IWM quasi-2D devices in N_2_ for long-term stability-test devices. **i** Normalized PCE variation curves of unsealed pristine, imprint, and IWM perovskite devices under continuous UV light in air

The stability of films against moisture, light and heat is a key factor in the commercialization of PSCs, so the stability tests of pristine, imprint and IWM films under the corresponding conditions are presented. The water contact angle of the imprint treated quasi-2D perovskite film was greatly improved to over 86° compared to the pristine, which can improve the humidity stability of the perovskite film and prevent degradation induced by water intrusion (Fig. S10). As proved in our previous work, hydrophobic (1H,1H,2H,2Hheptadecafluorodecyl) silane (FAS) oligomers as an anti-adhesive layer will partly move to the perovskite film during the imprint process to improve the hydrophobicity of the film [37]. Here, energy dispersive X-ray spectroscopy (EDS) (Fig. S11) shows that F and Si (characteristic elements in FAS) distributed over the entire IWM perovskite film, which indicates that part of the anti-adhesive layer is transferred to the film surface after imprint process. The water resistance of the pristine, imprint and IWM perovskite films was tested at 25 °C with 65% relative humidity (RH) in air. The variation of the absorption properties of pristine, imprint and IWM perovskite films further confirmed that the IWM perovskite films have better humidity stability than the pristine perovskite films (Fig. 4a–c). The absorption intensity of the pristine perovskite films decreased significantly compared to the IWM perovskite film, indicating that the pristine quasi-2D perovskite films underwent considerable degradation in high humidity environment. In contrast, IWM films still retained 80% of the original absorption after 600 h. Imprint can suppress the generation of grain boundaries, thus blocking the passageway of water intrusion, and leading to significantly enhanced humidity stability of quasi-2D perovskite film. In addition, images of pristine, imprinted and IWM film were recorded under continuous aging conditions of 55%–65% RH at 25 °C in air (Fig. S12), which further confirmed the improvement of humidity stability of imprint and IWM films. Figure 4d–f shows the XRD pattern corresponding to the aged films at 85 ℃ with 55% RH in air. Even after 300 h, the aged IWM perovskite films only showed a weak peak of lead iodide and a slight decrease in the intensity of the perovskite peaks, which indicate that the IWM treatment can greatly improve the environmental stability of quasi-2D perovskite film in air. The improved thermal stability and humidity stability of the films benefit from not only the hydrophobicity of large organic cations, but also the imprint process, which can effectively reduce the generation of grain boundaries to reduce the intrusion of moisture. Besides, the tight lattice structure enables IWM films to maintain good stability even at high temperatures. Meanwhile, UV–vis absorption spectra of pristine, imprint and IWM devices under continuous UV light was recorded in Fig. S13. After being irradiated by UV light for 120 h, the light absorption of pristine film decreased severely and imprint film decreased to some extent. In contrast, the IWM film exhibited best stability with no obvious change in the light absorption under continuous UV irradiation, because denser crystal structure of IWM film prevents further amplification of defects under UV light.

Based on the above stability test of quasi-2D perovskite film, so the stability tests of corresponding devices under various conditions were further assessed. Figure 4g shows the normalized PCE variation curves of unsealed pristine, imprint, and IWM devices which were aging in air at 25 °C with 55–65% RH. The stability of the pristine device of is the worst during the humidity aging process, with obvious PCE decease after 1,000 h. While the IWM device exhibited best stability with above 80% of initial PCE being retained. This is because imprint can suppress the generation of grain boundaries, thus blocking the passageway of water intrusion, leading to significantly enhanced humidity stability of PSCs. To understand the performance and stability of the perovskite devices with different n values, the stability of the devices with different n values was tested under this condition as shown in Fig. S14. The PCE of the devices increased as the n value increased, but the stability decreased compared to the devices with low n values, which was caused by the decrease in the content of large organic cations, but even so the devices with n = 50 still exhibited good stability. Furthermore, the stability of pristine, imprint, and IWM devices at 25 °C in nitrogen environment (Fig. 4h) were tested and the variation curves of PCE were recorded. After 2200 h, the degradation of the pristine device was serious with PCE decreasing to 0, while the IWM device present much better stability with ~ 82% of initial PCE being retained after 2,400 h. A comparison of perovskite solar cells based on 2D perovskite with high n value was shown in Table S2. Compared with other literatures, our work has obvious advantages in stability. In addition to humidity stability, UV stability of the devices was further tested by storing unsealed devices under continuous UV light irradiation in air (Fig. 4i). After 40 h, the pristine devices underwent degradation with ~ 50% of initial PCE being lost, while IWM device only lost ~ 20%, proving IWM device had better photostability. After IWM treatment, widely and uniformly distributed spacer cations may exist at the grain boundaries to suppress ion migration at the grain boundaries, thus effectively improving the photostability the device. Besides, the continuous performance of the unencapsulated PSCs was also examined by MPPT under 1 sun illumination in an N_2_ atmosphere (Fig. S15a). The IWM device exhibited enhanced light stability, maintaining 93% of its original PCE after 6,000 min, outperforming the pristine device, which degrades to 78% of the initial PCE. Our results represent the excellent stability of PSCs passing industry-relevant damp-heat test according to the ISOS-D-2 protocol. The IWM device after > 200 h of damp-heat test represented the best stability (Fig. S15b).

## Conclusion

In summary, MAAc and imprint were employed to optimize the phase distribution and improve the film quality of 2D perovskite successfully. The ionic liquid MAAc inhibited the aggregation of colloids in the precursor solution effectively, and thus further promoted the recrystallization in the process of imprint. Meanwhile, the imprint facilitated confinement crystallization and phase rearrangement in quasi-2D perovskite, leading to the sufficiently transition from low-n phase to 3D-like phase in quasi-2D perovskite. The abundant 3D-like phase with low n-phase is uniformly distributed within the perovskite, forming a bridge of charge transport throughout the film. Eventually, the efficiency of the quasi-2D device with IWM film reached to 18.96%, and corresponding devices exhibited improved thermal stability and humidity stability.

### Supplementary Information

Below is the link to the electronic supplementary material.

Supplementary file1 (PDF 1075 kb)
