# Supplementary material for: Ionic Liquid Assisted Imprint for Efficient and Stable Quasi-2D Perovskite Solar Cells with Controlled Phase Distribution
Source: Nanomicro Lett. 2023 Apr 7;15:91. doi: 10.1007/s40820-023-01076-8 (PMC10082145; doi:10.1007/s40820-023-01076-8)
Supplement: Supplementary file 1 — Supplementary file1 (PDF 1075 kb) [file 40820_2023_1076_MOESM1_ESM.pdf]

Supporting Information for

Ionic Liquid Assisted Imprint for Efficient and Stable Quasi-2D Perovskite Solar Cells with Controlled Phase Distribution

Haibin Peng[1, #], Dengxue Li[2, #], Zongcai Li[2], Zhi Xing[2, 3], Xiaotian Hu[2, 4], Ting Hu[1, 4, *], Yiwang Chen[2, 3, 4, *]

[1] Department of Polymer Materials and Engineering, School of Physics and Materials Science, Nanchang University, 999 Xuefu Avenue, Nanchang 330031, P. R. China

[2] College of Chemistry and Chemical Engineering | Institute of Polymers and Energy Chemistry (IPEC), Nanchang University, 999 Xuefu Avenue, Nanchang 330031, P. R. China

[3] National Engineering Research Center for Carbohydrate Synthesis/Key Lab of Fluorine and Silicon for Energy Materials and Chemistry of Ministry of Education, Jiangxi Normal University, 99 Ziyang Avenue, Nanchang 330022, P. R. China

[4] Peking University Yangtze Delta Institute of Optoelectronics, Nantong 226010, P. R. China

[#] H. Peng and D. Li contributed equally to this work.

*Corresponding authors. E-mail: huting@ncu.edu.cn (Ting Hu); ywchen@ncu.edu.cn (Yiwang Chen)

Supplementary Figures and Tables

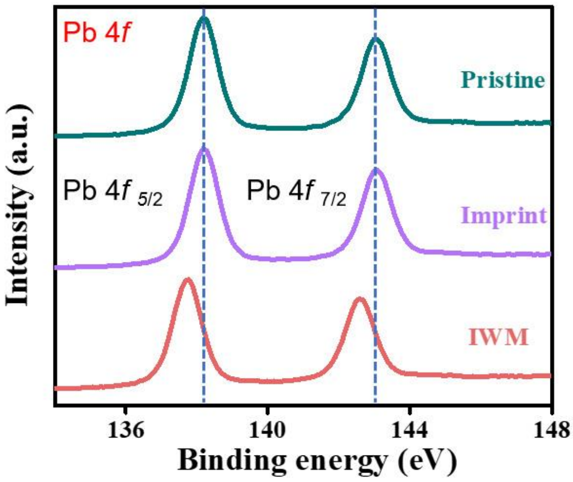

Fig. S1 XPS spectra of Pb 4f for pristine, imprint, and IWM films

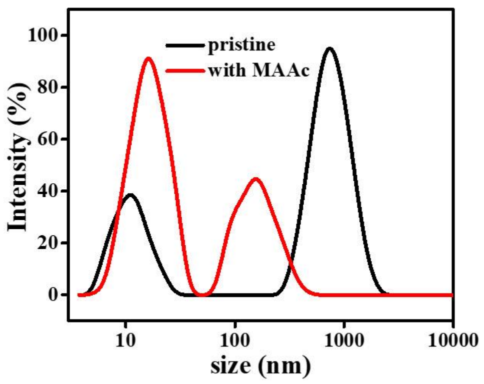

Fig. S2 Dynamic light scattering shows the particle size distribution of precursor solutions with and without MAAc

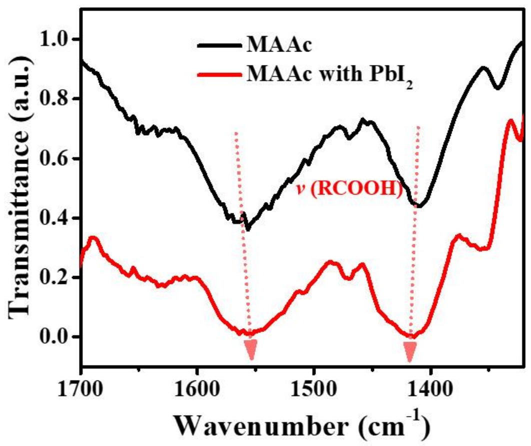

Fig. S3 The attenuated total reflection Fourier-transform infrared spectra of the MAAc and the solution prepared by dissolving PbI_2 in MAAc. The arrows indicate shifts of the asymmetric and symmetric stretching vibrations of CH_3COO^- upon interaction with Pb^{2+}

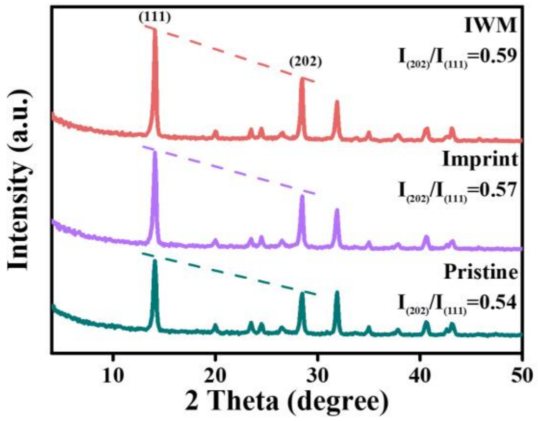

Fig. S4 XRD patterns of pristine, imprint and IWM perovskite films

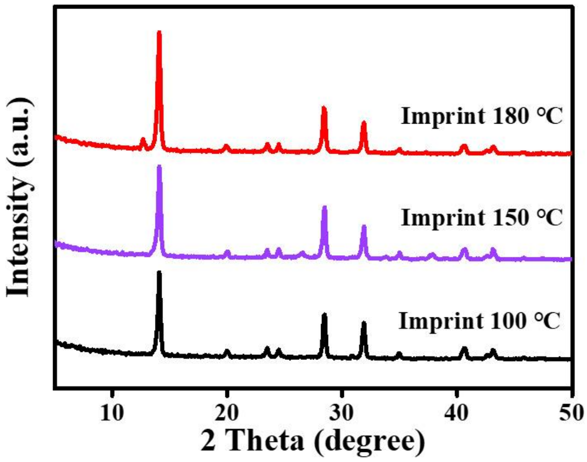

Fig. S5 XRD patterns of different imprinting temperatures

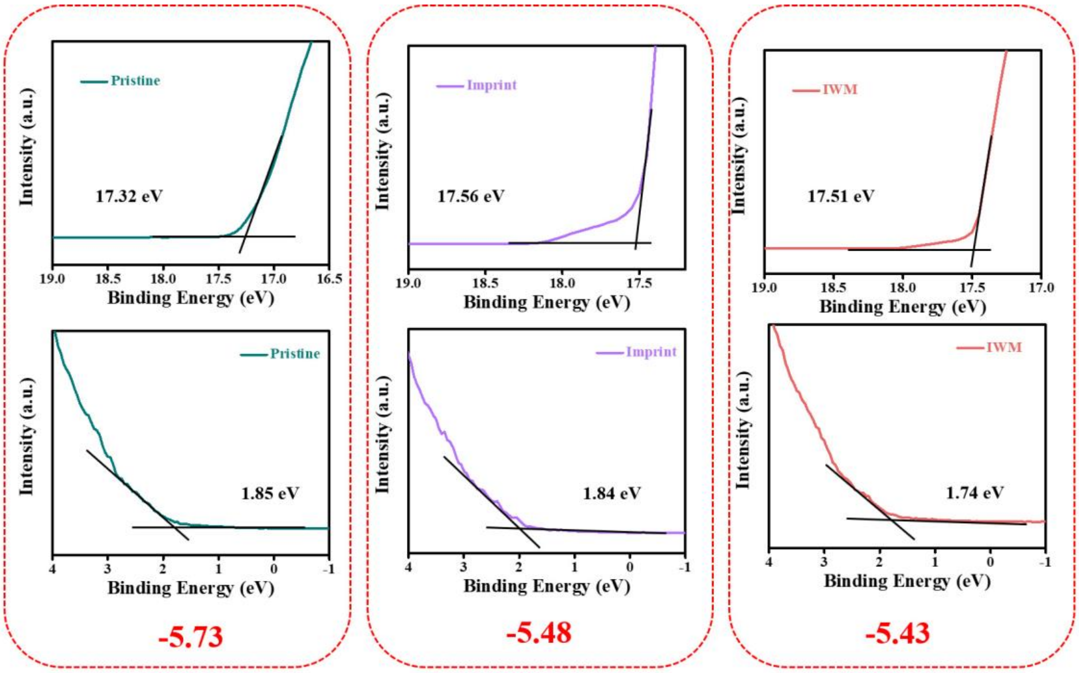

Fig. S6 The ultraviolet photoelectron spectroscopy (UPS) of pristine, imprint and IWM perovskite film

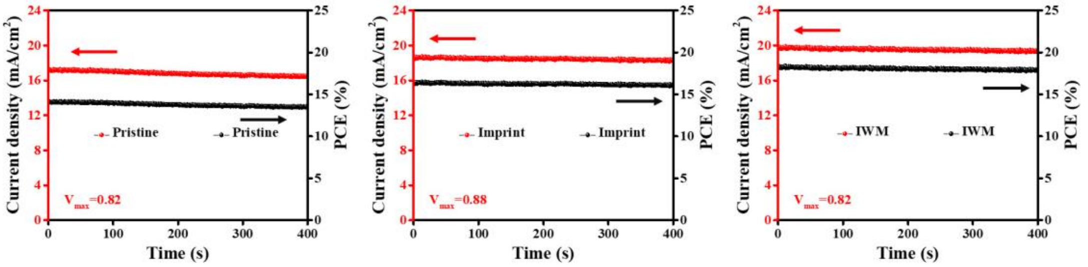

Fig. S7 Steady-state power output (SPO) and current density, measured for 400 s under the maximum power point (AM 1.5G, 100 mW cm^{-2}) at bias voltage recorded in the *J*–*V* curves

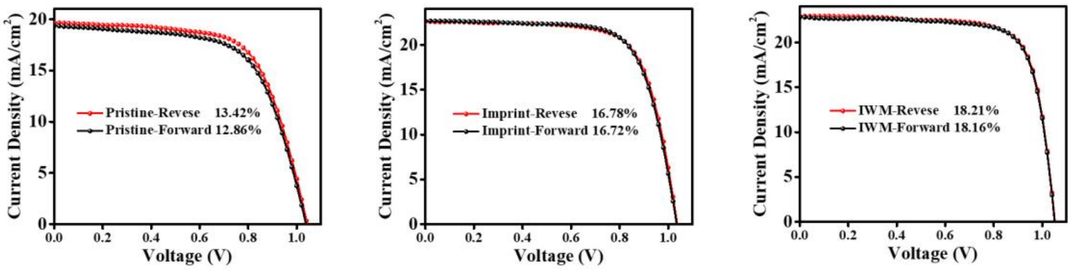

Fig. S8 *J*–*V* curves of pristine, imprint, and IWM (n=50) perovskite devices under reverse and forward scan directions

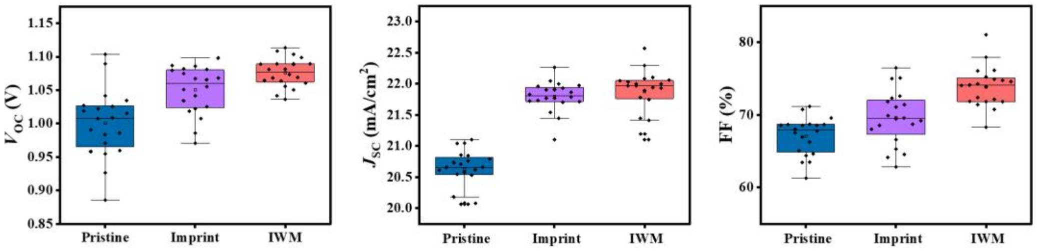

Fig. S9 V_{oc}, J_{sc} and FF distributions of pristine, imprint, and IWM perovskite devices

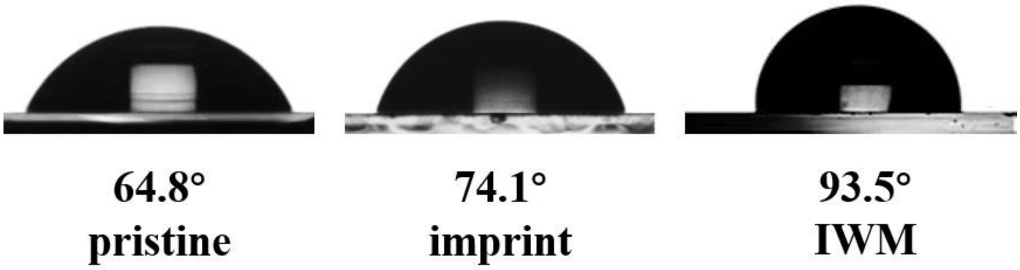

Fig. S10 The water contact angle of pristine, imprint, and IWM perovskite film

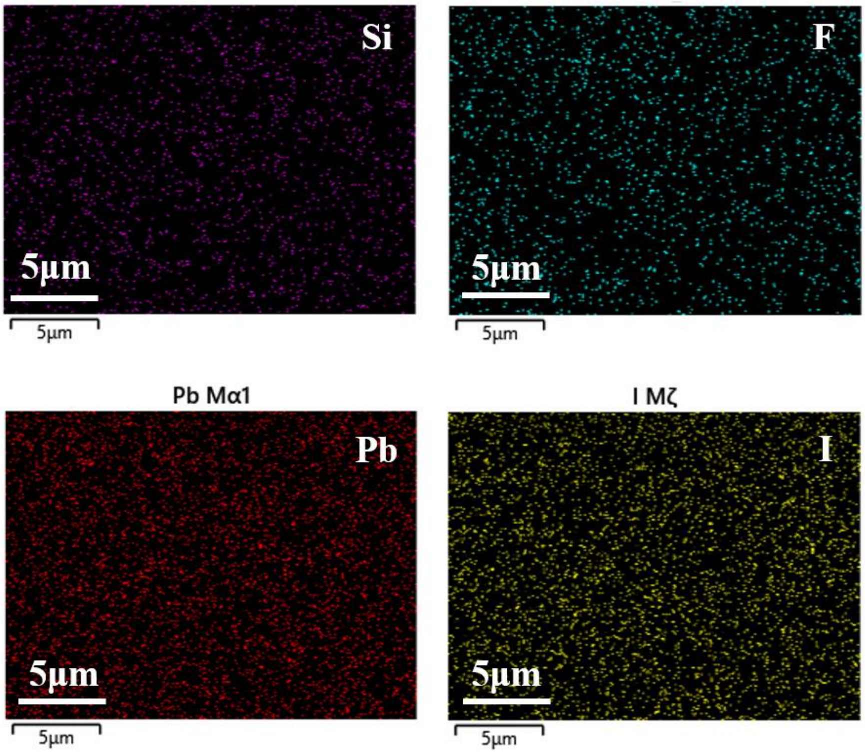

Fig. S11 SEM-EDS mappings of Si, F, Pb, and I elements for IWM perovskite film on ITO substrate

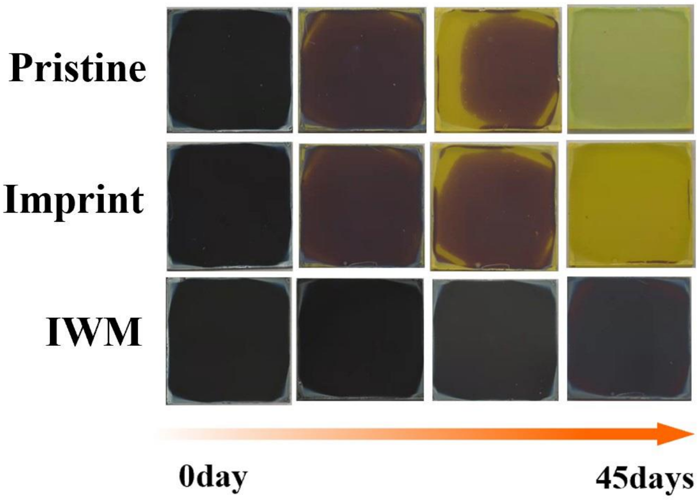

Fig. S12 Pictures of fresh and aged pristine, imprinted and IWM quasi-2D perovskite film by being stored in air with a relative humidity of 55–65% at 25 °C

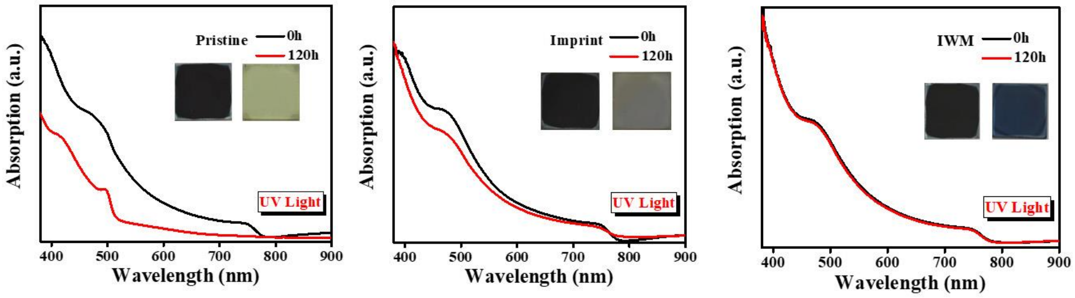

Fig. S13 UV absorption variation curves of unsealed pristine, imprint and IWM perovskite devices under continuous UV light

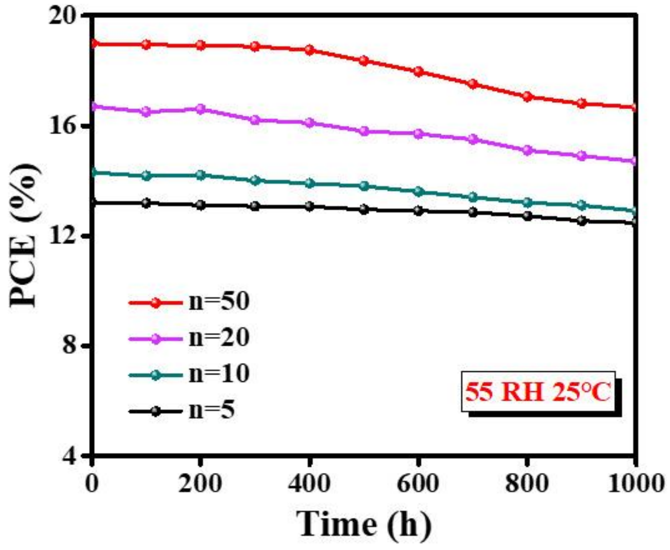

Fig. S14 PCE variation curves for unsealed devices with different n values in air at 25°C and 55% RH

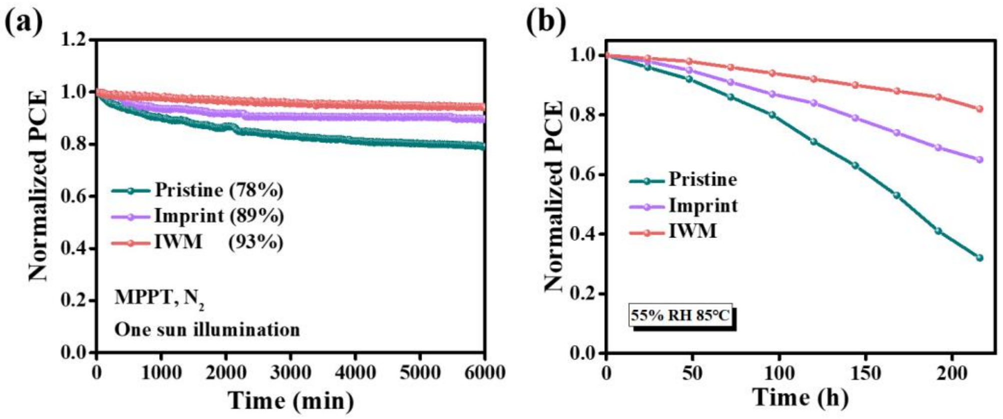

Fig. S15 a Continuous MPP tracking for the unencapsulated pristine, imprint and IWM devices under AM 1 sun illumination in N_2. **b** Variation of the PCEs at damp-heat test of pristine, imprint and IWM devices

Table S1 Comparison of efficiency of different phenylethylammonium-based 2D PVK

n value	PVK	Device structure	PCE (%)	Method	Refs.
5	$(4FPEA)_2(FA)_4Pb_5I_{16}$	(ITO)/ PTAA/2D RP perovskites/PCBM/ BCP/Ag	20.0	Component regulation and additives	[S1]
5	$(PEA)_2(MA)_4Pb_5I_{16}$	(ITO)/PEDOT:PSS /perovskites/PCBM/BCP/Ag	14.1	Additive strategy	[S2]
5	$4\text{-}BrPEA_2MA_4Pb_5I_{16}$	(FTO)/TiO_2/perovskites/2,2′,7,7′-Tetrakis[*N*,*N*-di(4-methoxyphenyl)amino]-9,9′-spiro-bifluorene (Spiro-OMeTAD)/MoO_3/Ag	15.2	Additive strategy	[S3]
5	$(PEA)_2(MA)_4Pb_5I_{16}$	ITO/PTAA/2D RP perovskites /$PC_{61}BM$/PEI/Ag	18.04	Vacuum Poling	[S4]
60	$(PEA)_2(Cs)_{59}Pb_{60}I_{18}1$	(ITO)/SnO_2/2D RP perovskites/spiro-MeOTAD/Au	12.4	Component regulation	[S5]
50	$(PEA)_2(MA)_{49}Pb_{50}Br_{151}$	(FTO)/B.L/TiO_2/2D RP perovskites/ spiro-MeOTAD/Au	8.5	Component regulation	[S6]
**50**	**$(PEA)_2(MA)_{49}Pb_{50}I_{151}$**	**(ITO)/SnO_2/2D RP perovskites/spiro-MeOTAD/Ag**	**18.9**	**Imprint and additives**	**This work**

Table S2 Stability performance of high n-value 2D perovskites in the literature

general formula	n values	device architecture	PCE (%)	stability (retained PCE, time, condition)	Refs.
$(PEA)_2(MA)_{39}Pb_{40}I_{121}$	40	Au/Spiro-OMeTAD/pvk/TiO_2/FTO	16.47	92.2% of PCE after 1344 h, in the N_2 environment	[S7]
$(C_6H_5CH_2NH_3)_2(FA)_8Pb_9I_{28}$	9	Au/Spiro-OMeTAD/pvk/m-TiO_2/bl- TiO_2/FTO	17.4	80.0% of PCE after 500 h, in the 80% relative humidity	[S8]
$(PEA)_2Cs_{39}Pb_{40}I_{121}$	40	Au/Spiro-OMeTAD/pvk/SnO_2/ITO	11.3	93% of PCE after 960 h, in the ambient atmosphere	[S5]
$4TFBZA_2MA_{59}Pb_{60}I_{181}$	60	Ag/Spiro-OMeTAD/pvk/SnO_2/ITO	17.07	84% of PCE after 1080 h, in the N_2 environment	[S9]
**$(PEA)_2(MA)_{49}Pb_{50}I_{151}$**	**50**	**(ITO)/SnO_2/2D RP perovskites/spiro-MeOTAD/Ag**	**18.9**	**~82% of PCE being after 2400 h. at 25°C in N_2 environment**	**This work**

Supplementary References

[S1]M. Shao, T. Bie, L. Yang, Y. Gao, X. Jin, F. He, N. Zheng, Y. Yu, and X. Zhang, Over 21% efficiency stable 2D perovskite solar cells. Adv. Mater. **34**, 2107211 (2022). https://doi.org/10.1002/adma.202107211

[S2]W. Fu, J. Wang, L. Zuo, K. Gao, F. Liu et al., Two-dimensional perovskite solar cells with 14.1% power conversion efficiency and 0.68% external radiative efficiency. ACS Energy Lett. **3**, 2086 (2018). https://doi.org/10.1021/acsenergylett.8b01181

[S3]D. Liang, C. Dong, L. Cai, Z. Su, J. Zang et al., Unveiling crystal orientation in quasi-2D perovskite films by in situ GIWAXS for high-performance photovoltaics. Small **17**, 2100972 (2021). https://doi.org/10.1002/smll.202100972

[S4]J. Zhang, J. Qin, M. Wang, Y. Bai, H. Zou et al., Uniform permutation of quasi-2D perovskites by vacuum poling for efficient, high-fill-factor solar cells. Joule **3**, 3061 (2019). https://doi.org/10.1016/j.joule.2019.09.020

[S5]Y. Jiang, J. Yuan, Y. Ni, J. Yang, Y. Wang et al., Reduced-dimensional α-$CsPbX_3$ perovskites for efficient and stable photovoltaics. Joule **2**, 1356 (2018). https://doi.org/10.1016/j.joule.2018.05.004

[S6]X. Li, K. Li, B. Wang, X. Zhang, S. Yue et al., Efficient and stable quasi-2D perovskite solar cells enabled by thermal-aged precursor solution. Adv. Funct. Mater. **31**, 2107675 (2021). https://doi.org/10.1002/adfm.202107675

[S7]L. N. Quan, M. Yuan, R. Comin, O. Voznyy, E. M. Beauregard et al., Ligand-stabilized reduced-dimensionality perovskites. J. Am. Chem. Soc. **138**, 2649 (2016). https://doi.org/10.1021/jacs.5b11740

[S8]H. Zheng, G. Liu, L. Zhu, J. Ye, X. Zhang et al., The effect of hydrophobicity of ammonium salts on stability of quasi-2D perovskite materials in moist condition. Adv. Energy Mater. **8**, 1800051 (2018). https://doi.org/10.1002/aenm.201800051

[S9]D. Li, Z. Xing, L. Huang, X. Meng, X. Hu et al., Spontaneous formation of upper gradient 2D structure for efficient and stable quasi-2D perovskites. Adv. Mater. **33**, 202101823 (2021). https://doi.org/10.1002/adma.202101823
